# Innovative Compact Vibrational System with Custom GUI for Modulating Trunk Proprioception Using Individualized Vibration Parameters

**DOI:** 10.3390/bioengineering12101088

**Published:** 2025-10-07

**Authors:** Debdyuti Mandal, John R. Gilliam, Sheri P. Silfies, Sourav Banerjee

**Affiliations:** 1 Department of Electrical and Computer Engineering, University of Southern California, Los Angeles, CA 90089, USA; 2 Pain Research and Intervention Center of Excellence, University of Florida, Gainesville, FL 32611, USA; johngilliam@ufl.edu; 3 Laboratory for Rehabilitation Neuroscience, Department of Applied Physiology and Kinesiology, University of Florida, Gainesville, FL 32611, USA; 4 Spine Neuromechanics Laboratory, Department of Physical Therapy, University of Delaware, Newark, DE 19713, USA; ssilfies@udel.edu; 5 Department of Mechanical Engineering, University of South Carolina, Columbia, SC 29208, USA

**Keywords:** vibrational system, proprioception, kinesthetic illusion, actuator, spinal injury

## Abstract

Conventional vibrational systems associated with proprioception are mostly equipped with a single standard frequency and amplitude. This feature often fails to show kinesthetic illusion on different subjects, as different individuals respond to different frequencies and amplitudes. Additionally, different muscle groups may also require the flexibility of frequencies and amplitudes. We developed a custom vibrational system that is equipped with flexible frequency and amplitude, adapted to a custom graphical user interface (GUI). Based on the user’s criteria, the proposed vibrational system enables a wide range of frequencies and amplitudes that can be swept under a single platform. In addition, the system uses small linear actuators that are wearable and attach to the subject without the need for restrictive straps. The vibrational system was used to model trunk proprioceptive impairment associated with low back pain. Low back pain is the leading cause of disability worldwide. It is mostly associated with impaired postural control of the trunk. For postural control, the somatosensory system transmits proprioceptive (position sense) information from the sensors in the skin, joints, muscles, and tendons. Proprioceptive studies on trunk muscles have been conducted where the application of vibration at a set amplitude and frequency across all participants resulted in altered proprioception and a kinesthetic illusion, but not in all individuals. To assess the feasibility of the system, we manipulated the trunk proprioception of five subjects, demonstrating that the vibrational system is capable of modulating trunk proprioception and the value of customizing parameters of the system to obtain maximal deficits from individual subjects.

## 1. Introduction

Human postural control relies on three feedback systems: visual, vestibular, and somatosensory. The somatosensory system transmits proprioceptive, or position sense, information from sensors in the skin, joints, tendons, and muscles. This information is used to update a predictive model of the body’s position in space, both relative to itself and the external environment [1,2,3]. Impaired proprioception has been established as an important factor predisposing individuals to injuries such as anterior cruciate ligament (ACL) rupture and lateral ankle ligament sprains [4,5,6,7,8]. Proprioceptive deficits are also hypothesized to be causally related to chronic low back pain (LBP) [9,10,11,12]. While not an exhaustive list of the conditions impacted by impaired proprioception, these injuries represent an enormous economic burden on healthcare systems and result in significant harm to the individuals afflicted [13,14,15,16,17,18]. Therefore, it is not surprising that researchers have sought to experimentally manipulate proprioception to elucidate the full scope of consequences associated with this impairment.

Proprioceptive information is transmitted to the central nervous system (CNS) primarily through cutaneous receptors, muscle spindles, and joint receptors. Muscle spindles are highly innervated, specialized muscle structures that provide proprioceptive information. Unlike joint receptors, which are most active at the end ranges of movement, muscle spindles are the position sensors most active in the middle ranges of motion [2]. These sensors are particularly relevant because, in most daily activities, we use our trunk (spine) within this middle range [1,2,3]. Trunk extensor muscles in the lower back demonstrate a relatively high density of muscle spindles [19,20]. Further, these same muscles exhibit characteristic structural and morphological changes in individuals with LBP, such as fatty infiltration and fibrosis that may compromise sensorimotor function [21,22,23,24,25].

Literature dating back several decades demonstrates that vibration applied to muscles results in increased discharge from primary muscle spindle afferent nerves, resulting in altered proprioception and a kinesthetic illusion where individuals perceive that the vibrated muscle is lengthening when in fact, it is not [26,27,28,29,30,31]. While muscle vibration predominantly activates muscle spindle afferents, vibration of the overlying skin and cutaneous receptors can also modulate the kinesthetic illusion [32,33]. The response to muscle vibration is consistent across different muscle groups, research paradigms, and study samples, including individuals with chronic LBP [28,34,35,36,37]. While this response is consistent, up to one-third of individuals in studies do not experience a kinesthetic illusion [37,38].

Understanding the role of impaired proprioception and its impact on movement and postural control will improve our understanding of the role of inaccurate regional muscle signals from this peripheral sensing system. A major limitation of previous trunk muscle vibration studies is the application of standard vibration parameters for all participants [10,36,39,40,41,42] when it is known that not everyone experiences a kinesthetic illusion at the same vibration frequency and amplitude [38,43]. Moreover, the most common vibration parameters used in previous investigations (frequency: 80 Hz, amplitude: 0.5 mm) of trunk extensor muscles are based on studies that vibrated only arm muscles, despite known differences in structure and function between these muscle groups [28,29]. Previous studies attempting to modulate trunk extensor muscle proprioception used vibration systems that operate using a DC motor and only generate discrete frequencies at a set amplitude [10,38,39,44]. This prohibited individualizing vibration parameters for participants. Additionally, previous work modeling trunk extensor muscle impairment has used belts to secure muscle vibrators to trunk muscles [10,38,39,44]. A belt that wraps around the trunk provides the participant with additional proprioceptive information through the contact of the belt with the skin and its cutaneous receptors, and inconsistently alters the preloading to the tissue, therefore injecting potential confounding variables into the experiment [45,46].

The pursuit of this novel muscle vibration device was driven by the desire to optimize a research paradigm to investigate the impact of impaired trunk extensor muscle proprioception on postural control. Commercially available products such as the Hypervolt (Hyperice; Irvine, CA, USA) and the Theragun (Therabody; Dallas, TX, USA) offer one to five discrete frequency settings with a uniform amplitude. These products are further limited because they operate at frequencies and amplitudes inconsistent with those used in the previous literature, inducing kinesthetic illusions and proprioceptive impairments. Additionally, these devices are relatively heavy and cannot be easily attached to the trunk without providing additional proprioceptive information through the skin. Researchers investigating the role of altered trunk proprioception in low back pain have used custom-made vibration systems that operate using a DC motor, which only generates discrete frequencies and amplitudes [9,10,38,39,44]. However, researchers investigating other body regions have developed custom systems that manipulate preload, frequency, or amplitude [46,47].

It would greatly improve current back pain research paradigms to have vibration systems that allow for finely tuned and personalized vibration parameters that can be adjusted for different trunk muscles, body types, and experimental paradigms. A vibration system that accounts for the between-individual variation in response to vibration parameters would help increase scientific understanding of trunk proprioceptive impairment and enable the creation of more effective back pain prevention and treatment strategies to address this societal need.

Based on these facts, there is a clear need for a vibrational system that is portable, accessible (unlike larger and heavier DC vibrators), has minimal metal components, and is easy to attach over varying muscle groups, with our particular interest being its use over human low back muscles. Inspired by these challenges, we present the development of a custom, compact, and portable vibrational system featuring small, lightweight actuators that can be attached to various body regions. The custom vibrational device is developed and equipped with variable frequencies and amplitudes so that the user has the flexibility to manipulate both quantities based on need. The proposed vibrational system is equipped with a customized Graphical User Interface (GUI) for controlling vibrational features, such as signal type, amplitude, frequency, phase, delay time, channel, and offset, thereby enhancing the quality of ease of operation. An example of its application to modeling maximum deficits in trunk proprioception across subjects demonstrates its feasibility.

## 2. Materials and Methods

**Figure 1:**
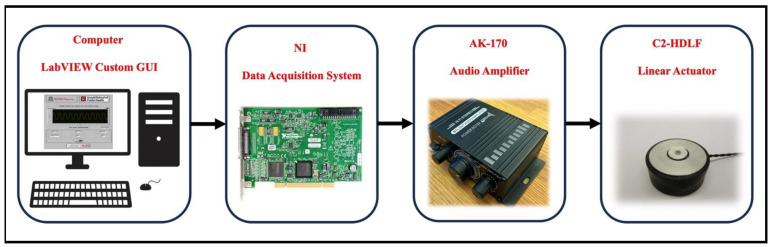
Schematic of the vibrational system.

The vibrational system consists of four major components: LabVIEW custom GUI, NI-DAQ, Audio Amplifier, and linear vibrational actuators. Figure 1 depicts the overall architecture of the vibrational system along with its components.

### 2.1. Custom LabVIEW Graphical User Interface (GUI)

**Figure 2:**
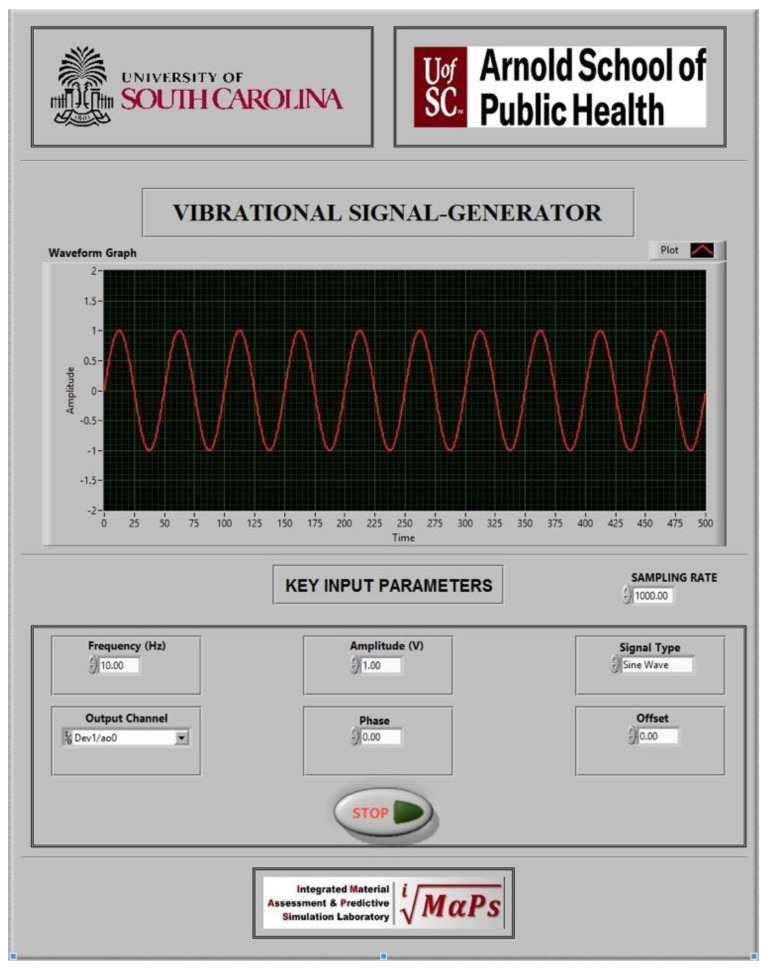
Custom GUI developed in LabVIEW for the vibrational system.

The initial phase of vibrational system development focused on designing a graphical user interface (GUI) tailored to user-defined requirements. The interface was implemented using NI-DAQ system integrated with LabVIEW software to ensure precision and functionality. Addressing the limitations of conventional vibrational systems, multiple parameters were integrated, resulting in a comprehensive and user-centric GUI design. Figure 2 depicts the custom GUI developed on the LabVIEW v8.6 software.

The GUI involves customized crucial parameters such as frequency, amplitude, type of signal, output channel for the DAQ, phase, offset, sampling rate, and delay time. The initial node in the system is the ‘create channel,’ which establishes the output channel to generate voltage signals for the vibrational system. This node is configured by assigning the output terminal, where users specify the desired output port based on their requirements. Following this, the ‘sampling clock’ node is connected to control the sampling rate and the number of samples to acquire or generate. A sampling rate of 1200 Hz was implemented for this system. The subsequent node, the ‘start trigger,’ interfaces with the sampling clock to configure tasks to begin acquiring or generating signals based on the rising or falling edge of a digital signal. A manual trigger mechanism is implemented within an if/else loop, allowing task execution to proceed when the condition is true and terminate otherwise. Once triggered, the program transitions to the ‘start task’ node, which shifts the task into the running state, initiating the acquisition or generation process. Following the configured delay after the trigger, the ‘start’ node initiates the program execution.

The system then advances to the ‘write’ node, which writes one or more floating-point samples to a task consisting of a single analog output channel. This process is embedded within a for-loop, which also contains the ‘basic function generator’ node. The function generator node produces various waveform signals, offering flexibility in parameters such as amplitude, frequency, phase, offset, and signal type. This configuration enables users to refine waveform signals tailored to specific requirements. Notably, the function generator node overcomes the limitations of conventional vibrational systems, which are typically restricted to a fixed amplitude and frequency. This advanced functionality plays a critical role in enhancing the versatility and capability of the vibrational system.

A ‘waveform’ node is integrated with the function generator node to visually monitor the user-defined waveform for the vibrational system on the interface. Changes in waveform parameters such as frequency, type, amplitude, phase, or offset are dynamically displayed, providing real-time confirmation of the generated signal. The primary nodes, namely the ‘write’ and ‘basic function generator’ nodes, are embedded within a for-loop, allowing the program to execute iteratively unless specified otherwise. To enhance control over the loop execution, an ‘elapsed time’ node is incorporated into the for-loop. This feature enables the loop to run for a user-defined duration, typically in seconds, providing autonomous control of the vibration system based on the specified time limit within the GUI.

**Figure 3:**
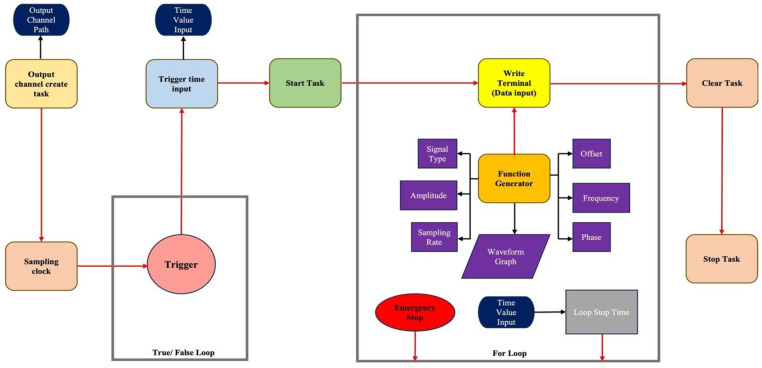
Custom Schematic block diagram of the whole customized vibrational system program circuitry and its components.

Following the completion of the specified duration, the program exits the for-loop and transitions to the ‘stop task’ node, which halts the overall operation and returns the task to its pre-execution state. Subsequently, the program advances to the final node, ‘clear task,’ which terminates the task, aborts any remaining processes if necessary, and releases the resources allocated to the task. Figure 3 illustrates the schematic representation of the LabVIEW program employed for the vibrational system.

### 2.2. NI Data Acquisition System

The data acquisition system consisted of two National Instruments M-series multifunction I/O data acquisition boards (NI-6229) with NI-DAQmx 9.5.1 driver software that interfaced with LabVIEW v8.6. Each NI-6229 device provides 16 differential analog inputs and 4 output channels at 16-bit ADC resolution with a sampling rate of 250 kS/s, CMRR 92 dB, 50 ppm timing accuracy, and 50 ns resolution. The maximum analog input voltage was ±11 V (signal + common mode) with an output voltage of ±10 V. Although the NI-DAQ serves a dual purpose for both data acquisition and actuation, this project solely used the actuation aspect for developing a custom function generator for the input of signals based on the user’s requirement for the vibrational system.

### 2.3. Signal Amplifier

The NI-DAQ produces significantly low power output, requiring a signal amplifier in the setup. In this study, the actual target was to develop a lighter and effective vibrational system. As a result, a simple audio amplifier was used instead of a bulky and expensive signal amplifier based on the linear actuator’s requirements. A Wemay AK-170 audio amplifier (Facmogu, Los Angeles, TX, USA) was used in the vibrational system as a signal amplifier. The audio amplifier system uses 2 channels with 20 W output. The device is equipped with a load impedance ranging from 4 to 160 Ω, with a signal-to-noise ratio of 90 db. The optimal frequency of the amplifier ranges from 20 Hz to 20 kHz. The device body incorporates an aluminum alloy body with a light and compact weight, quite ideal, unlike conventional heavy signal amplifiers, disrupting the intended purpose. RCA cables were used to connect the input channels with the NI-DAQ system.

### 2.4. Linear Actuators

The final components used in the vibrational system are the linear actuators, also referred to as tactors. As mentioned earlier, the trunk vibrational system uses bulky and big vibrators that are expensive and less portable. The most important reason is that bulky vibrators are very hard to fit into the human back. Additionally, in future investigations of proprioceptive impairments, usage of higher metal/magnetic components can lead to interference with the electromagnetic kinematic device (e.g., Polhemus), which is highly undesirable. Thus, addressing all of these issues, a simple linear actuator was incorporated for the purpose, which is cheaper and portable with minimal metal components. The primary reason for involving tactors is the need for a small and compact vibrational system allowing ease to attach to the lower back of the subject with medical tape, versus belting larger vibrators to the trunk, resulting in increased sensory input, restricted motion, and discomfort to the subject for the full proprioceptive study.

**Figure 4:**
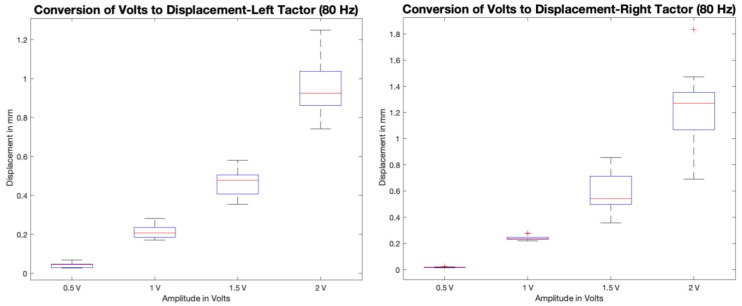
Calibration plots of C2-HDLF left and right tactors at 80 Hz. Voltage vs. displacement (in mm).

For this system’s development, C2-HDLF linear actuators were used. The C2-HDLF linear actuators were purchased from Engineering Acoustics, Inc., Casselberry, FL, USA. The operating frequency of the tactors ranges from 50 to 160 Hz, which lies in the range for proprioceptive study. The tactors possess maximum peak-to-peak displacement when loaded, ranging from 1.2 ± 0.052 mm. The linear actuators are compact, with each tactor weighing up to 30 g with a diameter of 1.2 inches, thus enabling it to be quite portable and friendly to wear. We utilized a high-accuracy vibration sensor meter to test the actual displacement of the left and right tactors and to demonstrate the actual relation of voltage vs. displacement. Ten samples were taken, corresponding to each voltage value ranging from 0.5 V to 2 V with an interval of 0.5 V. Figure 4 demonstrates the displacement vs. applied voltage calibration at 80 Hz (both left and right tactors).

## 3. Experimental Test Capabilities

**Figure 5:**
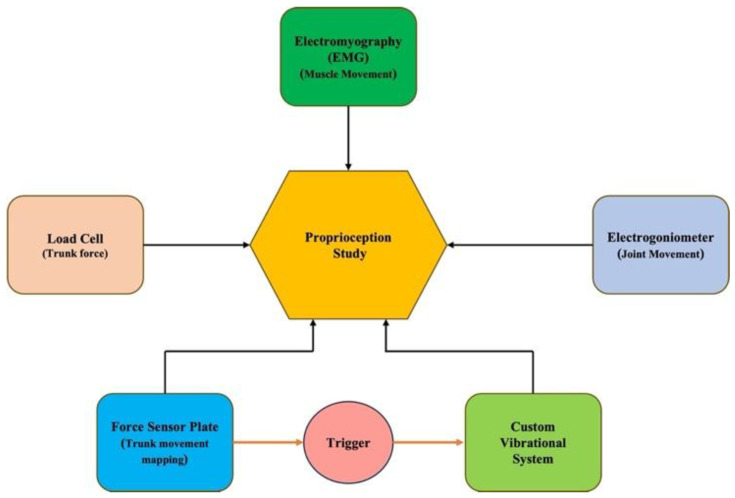
Schematic block diagram of the full capabilities for inducing proprioceptive alteration setup and its components.

For proprioceptive studies, the system allows configuration of several components. The software can incorporate the synchronized collection of electromyography, kinematics/goniometers, a single-axis load cell, and a force platform, allowing full capture of muscle activation and segmental movement. All these devices and their collection parameters are specifically controlled under a single LabVIEW program. For the following example of the system’s use for modulating proprioception of the lumbopelvic region, a dual-axis electrogoniometer was used for measuring trunk motion/position, and the vibration system was utilized for inducing vibrational actuation to the lower back of the subject. The program that controls the vibrational parameters is integrated into the main data collection program, with the trigger terminal acting as a bridge between the sensors and the vibrational program. Figure 5 shows the schematic block diagram of the full capabilities of the system for studying proprioceptive impairment.

### 3.1. Linear Experimental Setup for Inducing Proprioceptive Alteration in the Lumbopelvic Region

**Figure 6:**
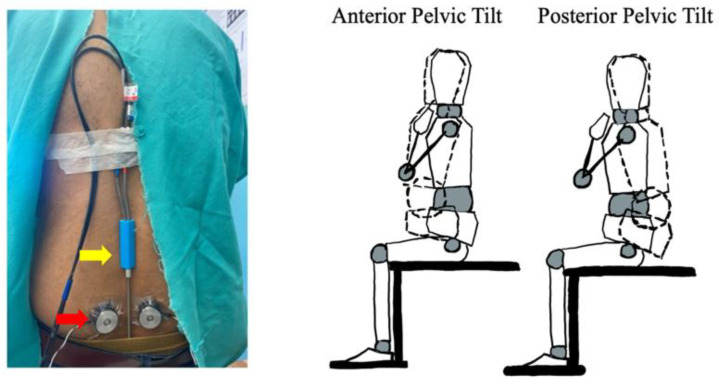
Experimental setup (left) demonstrates the arrangement of the vibrators (red arrow) and the electrogoniometer (yellow arrow). Illustration of anterior and posterior pelvic tilt motion relative to neutral seated posture and the associated reconfiguration of the trunk and pelvic segments (dashed lines) is represented on the right.

Pilot testing of the system on five human subjects was carried out under an IRB-approved protocol (Pro00124463). Vibrators were applied to the left and right lumbar erector spinae muscles of back-healthy controls two centimeters lateral to the fourth lumbar spinous process (Figure 6, red arrow).

Care was taken to ensure the vibrators were applied directly over the muscle belly and not over the bone. Tegaderm and tape were used to secure the vibrators, which removed the need for a belt that wraps around the trunk. Lumbopelvic joint position was quantified using a 2D electrogoniometer (Noraxon USA, Inc., model 508, Scottsdale, AZ, USA) secured with double-sided tape at the spinous process of L1 and on the sacrum at the level of S2 (Figure 6, yellow arrow).

### 3.2. Linear Active Joint Reposition Error Testing Paradigm

**Figure 7:**
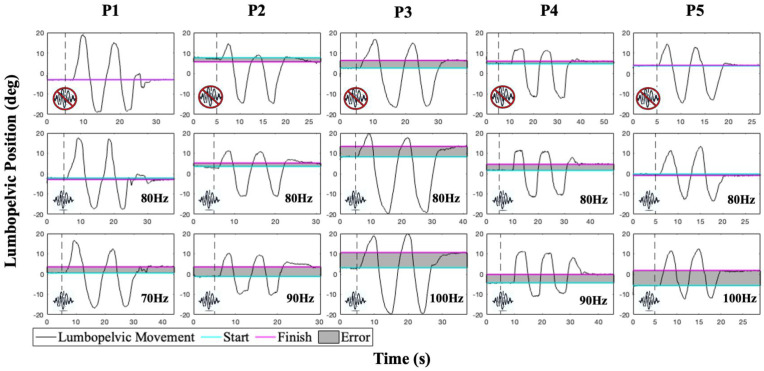
Example time series plots of active joint reposition error testing for five participants. The cyan line represents the starting position for each trial, the pink line indicates the final position for each trial, and the gray area represents the difference between the two (error). Top row: baseline tests without vibration. Middle row: 80 Hz vibration, standard in the trunk muscle vibration literature. Bottom row: vibration frequency producing a maximum repositioning error for each participant. Of note, the frequency that induced the maximum error differed across participants.

Participants performed lumbopelvic active joint reposition error (AJRE) testing while seated and blindfolded [9,11,48]. Verbal cues were provided to elicit movement from the lumbar spine and pelvis through anterior and posterior pelvic tilting (Figure 7). The movement performed was transitioning from anterior pelvic tilt (tilting the pelvis forward and arching the back, such as sitting upright with a posterior concavity of the lumbar spine) to posterior pelvic tilting (seated slouching). This motion was chosen as it isolates lower back motion, represents middle range trunk motion, and is accomplished by concentric and eccentric activation of the trunk extensor muscles. At the start of each baseline trial, subjects were verbally guided by a researcher to a target position within the midrange of their full lumbopelvic motion by using real-time feedback from the electrogoniometer. Once a subject achieved the target position, they held the position for 5 s before tilting their pelvis anteriorly and posteriorly through their full lumbopelvic range of motion two times. They then actively tilted their pelvis to match the target position (no verbal guidance) and were instructed to hold at the estimated target position for 5 s.

Subjects performed baseline trials of AJRE testing without vibration to determine baseline AJRE performance. Participants then completed AJRE trials with TEM vibration at frequencies of 70, 80, 90, and 100 Hz with a constant peak-to-peak amplitude of 0.5 mm [10,38,39,44,49]. Vibration frequencies were presented three times each in a pseudorandom and interleaved order to control for muscle spindle saturation and the potential cumulative effects of the vibration stimulus. Testing occurred in two blocks, with a 5 min rest period between blocks. Between the blocks, participants were allowed to move freely, as this allowed time for the lumbopelvic movement with accurate proprioceptive information. Before the second block, participants performed 3 trials of AJRE testing without vibration to ensure washout of vibration effects. Within each trial, the vibration stimulus began after the guided target position was held for 5 s and continued until the end of each trial (after the estimated target position was held for 5 s). Target position (lumbopelvic angle) for each AJRE trial was calculated as the mean of the first 3 s of data collection. The lumbopelvic angle associated with repositioning at the target was calculated as the mean of the final 3 s of data. Errors in each AJRE trial were calculated as the difference, in degrees, between the target and reposition angles.

## 4. Results and Discussion

Demographic data for five back-healthy participants are presented in Table 1. Mean and standard deviation of each participant’s AJRE across the frequencies tested are presented in Table 2. The increase in AJRE between the baseline (no vibration) and the highest error ranged from 60% to 173%. Time series plots of AJRE testing for participants are presented in Figure 7. The first row in Figure 7 represents baseline AJRE testing performance without vibration. The second row displays AJRE testing with 80 Hz vibration, which is commonly used in trunk muscle vibration studies [2,39,50]. The third row exhibits the frequency that elicited the greatest error in each participant. The dotted lines in rows two and three signal the onset of the vibration stimulus. Participant 1 displayed the smallest error values, and Participant 3 had the largest differences between the start and finish positions. Despite differences in the amount of errors, each participant demonstrated an increase in error with vibration. We observed that the maximum AJRE across subjects was obtained at a different vibration frequency. Five back-healthy individuals displayed maximum lumbopelvic AJRE during three different vibration frequencies applied to the TEM. An 80 Hz frequency vibration, a common frequency used for muscle vibration in the literature, did not result in maximal active repositioning error in any of these participants.

These preliminary data suggest that applying a standard vibration frequency to all individuals may be suboptimal for inducing maximal proprioceptive alteration. Variability in the frequency that maximally induced lumbopelvic repositioning error is preliminary evidence of the value of a flexible custom vibration system. The flexibility to personalize vibration parameters to induce larger effects on proprioception may provide a better testing paradigm for understanding proprioceptive deficits reported in rehabilitation and sports medicine populations.

This flexible custom vibration system, integrated within a larger data collection system that allows for the simultaneous collection of goniometry, electromyography, kinematics, and force plate measures, provides a means to better understand the biomechanical consequences of impaired proprioception in a variety of populations. Further, the easy-to-use GUI allows researchers in rehabilitation and sports medicine domains to customize parameters for their specific purposes. This flexibility is ideal for application to a variety of research questions that include targeting different tissues or receptor types, testing paradigms to optimize deficits or illusions across and within different populations, muscle groups, or tasks to better understand proprioceptive deficits that contribute to prolonged movement impairments or reinjury. A study using our AJRE paradigm and vibration system to model proprioceptive deficits in trunk extensor muscles and assess the impact on trunk postural control further demonstrates the utility of our vibrational system [51].Future experiments using an AJRE paradigm and our vibration system will focus on understanding why up to one-third of individuals do not experience a noticeable effect of muscle/tendon vibration and on modeling proprioceptive deficits and related movement impairments reported in individuals with musculoskeletal injuries.

While testing five subjects shows the feasibility of our system, more work is needed. To better understand the variation between individuals in response to vibration frequency, studies with larger sample sizes and inclusion of variations in vibration amplitude will be necessary. Additionally, to gain insight into the mechanisms behind individual differences, future research should investigate factors influencing responses to vibration. One example is measuring the subcutaneous tissue between the vibrator and the target muscle, as this tissue may reduce the vibrational signal. Another possible source of response variability could be the condition of the muscle tissue; fibrosis and fatty infiltration might affect muscle spindle function. Future studies could also examine the relative contributions of cutaneous receptors and muscle spindles in response to vibration applied to the trunk.

## 5. Conclusions

We developed a customized vibrational system equipped with the feature of variable frequency and amplitude based on the user’s requirements. A custom LabVIEW GUI was developed, having the freedom of different signal parameters, which is integrated into NI-DAQ systems for actuation. The system involves two independent linear actuators that are easily attachable to the human body without discomfort and interference, thus convenient for other proprioceptive equipment interaction. The system was developed for and tested on real human subjects for feasibility and preliminary evidence that, in different individuals, frequencies targeting muscle spindles may need to be individualized to achieve maximum modulation of proprioception acuity.

## Figures and Tables

**Table 1:** Data for 5 participants having a healthy back.

Participant Demographics					
	P1	P2	P3	P4	P5
Age (years)	26	23	22	33	31
Sex	F	M	F	M	F
BMI (kg/m^2^)	20.9	25.6	19.0	23.6	23.6
AROM (degrees)	28.4 (0.8)	27.2 (0.2)	33.8 (2.1)	47.5 (0.3)	42.5 (1.4)

P: participant; BMI: body mass index; F: female; M: male; AROM: active range of motion between maximum anterior and posterior pelvic tilt.

**Table 2:** Absolute error across vibration frequencies.

Mean (SD) Absolute Error with AJRE Testing					
	P1	P2	P3	P4	P5
Baseline	1.0 (1.1)	1.9 (1.0)	4.4 (0.6)	1.1 (0.8)	1.5 (1.1)
70 Hz	**2.2 (0.7)**	3.8 (4.5)	7.2 (3.1)	1.2 (1.5)	1.8 (0.9)
80 Hz	1.6 (1.9)	2.0 (1.8)	6.0 (3.9)	1.3 (0.9)	1.5 (0.6)
90 Hz	1.7 (1.3)	**4.9 (2.1)**	6.4 (6.0)	**3.0 (2.5)**	1.7 (0.7)
100 Hz	1.2 (2.0)	1.2 (1.2)	**8.6 (3.2)**	2.7 (1.7)	**2.4 (0.5)**

Results are presented as mean and standard deviation (SD) in degrees from target position. Baseline: no vibration condition; AJRE: absolute joint repositioning error; Hz: Hertz; **Bold**—highest mean error.

## Data Availability

The original contributions presented in this study are included in the article. Further inquiries can be directed to the corresponding author.
